# Prognostic significance of *SETBP1* mutations in myelodysplastic syndromes, chronic myelomonocytic leukemia, and chronic neutrophilic leukemia: A meta-analysis

**DOI:** 10.1371/journal.pone.0171608

**Published:** 2017-02-03

**Authors:** Li-Hong Shou, Dan Cao, Xiao-Hui Dong, Qiu Fang, Ying Wu, Yan Zhang, Ju-Ping Fei, Bao-Lian Xu

**Affiliations:** Department of Hematology, Huzhou Central Hospital, Huzhou, Zhejiang, China; Cardiff University, UNITED KINGDOM

## Abstract

**Objectives:**

This meta-analysis investigates the prognostic effect of SET binding protein 1 (*SETBP1*) mutations in patients with myelodysplastic syndromes (MDS), chronic myelomonocytic leukemia (CMML), or chronic neutrophilic leukemia (CNL).

**Methods:**

Eligible studies from Pubmed, Embase, and Web of Science were searched from database inception through April 2016. Hazard ratios (HRs) and 95% confidence interval (CI) of overall survival (OS) were pooled to calculate the prognostic significance of *SETBP1* mutation in patients.

**Results:**

A total of 12 studies with 2321 patients were included in this meta-analysis; 4 studies for MDS, 5 studies for CMML, and 3 studies for CNL. Pooled results suggested that MDS and CMML patients with *SETBP1* mutations had a significantly poorer prognosis when compared with patients with wild-type *SETBP1* (MDS: HR = 1.808, 95% CI (1.218–2.685), P = 0.001; CMML: HR = 2.223, 95% CI (1.493–3.308), P<0.001). *SETBP1* mutations in CNL patients however, showed no significant effect on the overall survival (HR = 1.773, 95% CI (0.877–3.582), P = 0.111). The Begg’s and Egger’s tests did not show significant publication bias in any groups.

**Conclusions:**

Current evidence shows that *SETBP1* mutation is associated with a poor prognosis in patients with MDS and CMML, but not in patients with CNL.

## Introduction

*SETBP1*, a gene located on chromosome 18q21.1, encodes SET binding protein 1 that binds with SET nuclear oncoprotein to form a heterodimer complex. Although incompletely characterized, *in vitro* studies have suggested that this heterodimer inhibits the activity of protein phosphatase type 2A (PP2A), a tumor suppressor, and increases the rate of cell proliferation [1, 2].

Germline mutations in *SETBP1* were first identified in children with Schinzel-Giedion syndrome (SGS), where *SETBP1* mutations caused congenital disorder characterized by mental retardation, facial deformities and congenital anomalies [3, 4]. More recently, studies have revealed the association of somatic *SETBP1* mutations with different types of leukemia. Cristobal *et al*. demonstrated that the t(12;18)(p13;q12) translocation induced the overexpression of *SETBP1*, leading to poor prognosis in elderly patients with acute myeloid leukemia (AML) [1]. In 2013, exome-sequencing analysis of patients with atypical chronic myeloid leukemia (aCML) identified the presence of missense *SETBP1* mutations [2]. Patients who had *SETBP1* mutations had poorer prognosis with a higher white blood cell count when compared with patients who had wild-type *SETBP1* [2]. These mutations were clustered from residue 858 to 871 located in the highly conserved SKI-homologous domain [3], suggesting a functional alteration in the mutations.

*SETBP1* mutations are also detected in myelodysplastic syndromes (MDS), chronic myelomonocytic leukemia (CMML), and chronic neutrophilic leukemia (CNL). For example, Piazza *et al*. detected *SETBP1* mutations in 3 out of 82 patients with CMML but no *SETBP1* mutations were found in 100 patients with MDS [2]. Makishima *et al*. detected *SETBP1* mutations in 22 out of 152 patients with CMML [5] and Damm *et al*. observed *SETBP1* mutations in 12 out of 195 patients with CMML and in 5 out of 222 patients with MDS [6]. Elliott *et al*. also detected *SETBP1* mutations in 5 out of 14 patients with CSF3R-mutated CNL [7]. Recently, through whole exome sequencing, Mason *et al*. identified *SETBP1* mutations in 11 out of 69 patients with CMML [8] and Merlevede *et al*. reported detection of *SETBP1* mutations in one out of 49 patients with CMML [9]. However, the prognostic significance of *SETBP1* mutations in MDS, CMML, and CNL remains inconclusive. Reports have suggested that the presence of *SETBP1* mutations in these myeloid malignancies reduced survival or are associated with poorer prognosis in patients [10, 11]. However, some studies have also reported that SETBP1 had no significant effect on the overall survival [12, 13]. Therefore, our meta-analysis aims to evaluate the prognostic effect of *SETBP1* mutations in patients with MDS, CMML, and CNL. To the best of our knowledge, this is first meta-analysis to evaluate the overall survival (OS) or hazard ratio (HR) for *SETBP1* mutations in these patients.

## Methods

### Literature search

Relevant literatures were systematically searched from electronic databases including PubMed, Embase, and Web of Science. The last search date was April 21, 2016. The following search terms were used: "SET binding protein 1" OR SETBP1 OR SEB AND (leukemia OR leukaemia OR leucocythaemia OR leukemia OR MDS OR "myelodysplastic syndrome" OR "myelodysplasia" OR "preleukemia" OR "myelomonocytic leukemia" OR acml OR “atypical chronic myeloid leukemia” OR “myeloid malignancies” OR “neutrophilic leukemia”) AND (prognosis OR survival OR prognostic OR "Kaplan-Meier analyses").

### Study selection

Studies were included when they met the following inclusion criteria: All published articles (including letter to editor) focused on *SETBP1* mutations in MDS, CMML, and CNL patients, and provided data on the overall survival (OS) or leukemia-free survival (LFS).

The exclusion criteria were as followed: (1) Articles on the association of *SETBP1* expression levels with the OS and LFS of patients with MDS, CMML and CNL; (2) Type of disease was not specified in the article; (3) Diseases other than MDS, CMML, CNL and aCML; (4) Conference proceedings that contained only summaries; (5) Literature published in languages other than Chinese or English; (6) Literature with data that could not be extracted or data that were duplicated.

### Data extraction

Two reviewers independently extracted the following information from the included studies: first author, year of publication, source of publication, types of diseases, population studies, total number of patients and number of patients with *SETBP1* mutation, diagnostic criteria for disease, mutation detection methods, types of *SETBP1* mutations and the corresponding hazard ratios (HRs) with 95% confidence interval (95% CI) for OS. When the HR values were not provided and the only available data were presented in the form of graphical survival curves, the Engauge Digitizer software version 4.1 was used to extract the X- and Y-coordinates of each event from the curve, and converted the coordinates into estimated survival rates at specified time points. SPSS19.0 was then used to reconstruct the Kaplan-Meier curve, and the HR estimates and 95% CIs were obtained using Cox regression analysis. For the remaining data that could not be extracted, contacts with the authors were made to fill in the missing data. Disagreements were resolved through consensus of a third reviewer.

### Methodological quality assessment

The methodological quality of the included studies was evaluated using the Newcastle-Ottawa Scale (NOS). The NOS consisted of 3 categories (Selection, Comparability, and Exposure/Outcome). The categories were assigned 4, 2, and 3 stars respectively, with the distribution of the stars as follows: In the Selection and Exposure/Outcome categories, a maximum of one star could be awarded for each numbered item within each category, whereas in the Comparability category, a maximum of two stars could be awarded for a high quality study. Therefore, the quality of an included study could range between 0–9 stars.

### Statistical analysis

All meta-analyses were performed using Stata software version 12.0 (College Station, TX, USA). The effects of *SETBP1* mutations on OS were assessed using overall HR and 95% CI. Heterogeneity between studies was evaluated using the Q test (where P<0.10 was considered significant) and I^2^ statistic (I^2^ = 0–25%, no heterogeneity; I^2^ = 25–50%, moderate heterogeneity; I^2^ = 50–75%, large heterogeneity; I^2^ = 75–100% extreme heterogeneity). When there was no statistically significant heterogeneity (I^2^<50%) present, fixed-effects model was adopted as the pooling method. Otherwise, the random-effects model was conducted. HR>1 indicated that patients with *SETBP1* mutations have a poorer prognosis than patients with wild-type *SETBP1*. The sensitivity analysis was performed using the sequential omission of individual studies to assess the quality and robustness of the results. Publication bias was assessed using Begg’s and Egger’s tests. A two-sided P value less than 0.05 was considered to be statistically significant.

## Results

### Study selection

**Fig 1:**
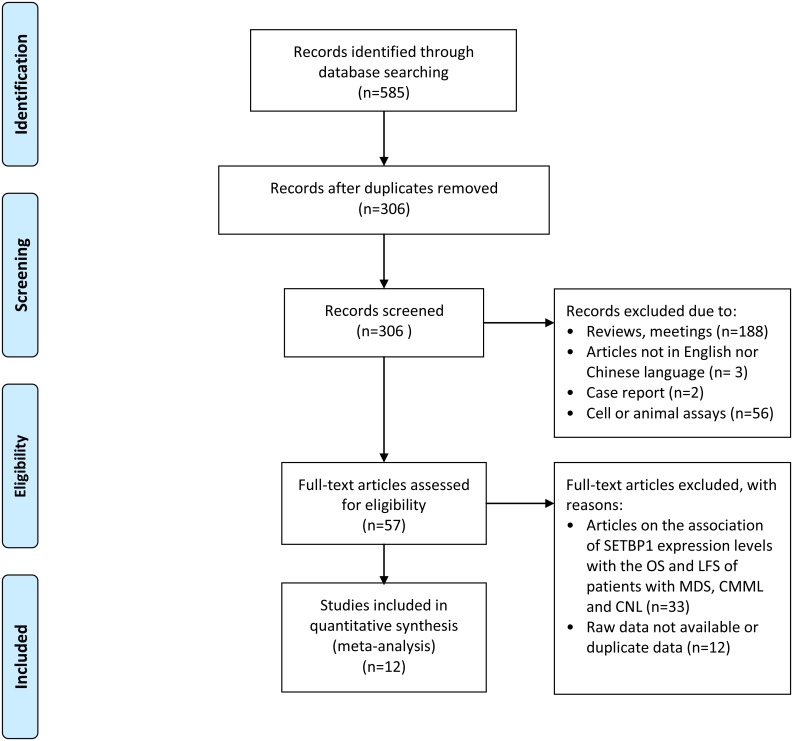
Flow chart of study selection.

Our systematic literature search identified a total of 585 publications and 279 were removed because they were duplicates. The remaining 306 publications were reviewed by their titles and abstracts, 249 articles were then eliminated due to the following reasons: reviews or meetings abstracts (188), articles neither in English nor Chinese (3), case reports (2), and articles related to cell or animal assays (56). Another 45 articles were excluded after full text review as they were articles on the association of *SETBP1* expression levels with the OS and LFS of patients with MDS, CMML and CNL or the raw data was not available or duplicated. This left 12 studies that met the criteria for this meta-analysis (Fig 1).

### Study characteristics

Twelve studies with a total of 2587 patients were included for meta-analysis; 1167 patients with MDS (4 studies), 1364 patients with CMML (5 studies), and 56 patients with CNL (3 studies). The characteristics and patient demographic data are summarized in Table 1. All studies examined patients with MDS, CMML, or CNL screened for *SETBP1* mutations and their corresponding OS. These studies were reported between 2013 and 2015. Of these studies, 4 originated from USA, 4 from Europe, and 4 from Asia. Sample sizes of these studies varied considerably, from as few as 8 patients to as many as 727 patients. Most of the diagnosed diseases were based on the WHO classification except for one study where the disease classification was not mentioned. Ten studies identified *SETBP1* mutations using PCR and Sanger sequencing; however, two studies did not mention the methods for detection.

**Table 1:** Summary of data extracted from 12 studies included in the meta-analysis.

Study	Year of publication	Journal	Types of diseases	Population studied	Patient (total)	Diagnosis criteria of disease	Rate of SETBP1 mutation case	Method to distinguish mutations	*SETBP1* mutations
A Pardanani [14]	2013	Leukemia	CNL	USA	35	WHO classification	17%	PCR and Sanger sequencing	D868N D870D G827R G870S
Daichi Inoue [15]	2015	Leukemia	MDS	Taiwan	386	WHO classification	9%	PCR and Sanger sequencing	D868N E858K G870S S867R
F Damm [6]	2013	Leukemia	CMML	France	195	WHO classification	6%	PCR and Sanger sequencing	D868N G870S G870D E858K D868H S869G S869R I871S D908N
F Thol [12]	2013	Leukemia	AML and MDS	German	326	WHO classification	2%	PCR and Sanger sequencing	D868N G870S S854A S869N
Hideki Makishima [5]	2013	Nat Genet	MM	Japan	727	WHO classification	-	PCR and Sanger sequencing	D868N G870S I871T D868T D880N S869N D880E
Hou [10]	2014	American Journal of Hematology	MDS	Taiwan	430	WHO classification	3%	PCR and Sanger sequencing	D868N G870S E858K S867R I871T
M Meggendorfer [16]	2013	Leukemia	CMML and aCML	Italy	227	WHO classification	12%	PCR and Sanger sequencing	G870S I871T G870D
Michelle A. Elliott [7]	2015	American Journal of Hematology	CNL	USA	13	WHO classification	31%	-	G870D D868N G872R
Mrinal M. Patnaik [17]	2015	American Journal of Hematology	CMML	USA	36	WHO classification	3%	-	-
RR Laborde [11]	2015	Leukemia	CMML	USA	179	-	4%	PCR and Sanger sequencing	D868N D868Y G870S I871T
Vera Adema [18]	2015	British Journal of Haematology	MDS	Barcelona	25	WHO classification	50%	PCR and Sanger sequencing	-
Cui [19]	2014	Chinese Journal of Hematology	CNL	China	8	WHO classification	50%	PCR and Sanger sequencing	D847N D868N G870S I871T

WHO: World Health Organization

MM: Myeloid Malignancies

CNL: Chronic neutrophilic leukemia

aCML: Atypical chronic myeloid leukemia

MDS: Myelodysplastic syndromes

CMML: Chronic myelomonocytic leukemia

### Quality of studies

Individual studies scored well on the NOS; 10 studies scored 8 out of 9 stars, 1 study scored 7 out of 9 stars, and 1 with 6 out of 9 stars (Table 2). Overall, all studies included in this meta-analysis were of high quality.

**Table 2:** Quality assessment of individual study.

Study	Selection				Comparability	Outcome			Score
	Representativeness of exposed cohort	Selection of non-exposed cohort	Ascertainment of exposure	Outcome not present at start		Assessment of outcome	Follow-up length	Follow-up adequacy	
**A Pardanani (2013)** [14]	*	*	*	*	*	*	*	*	**8**
**Daichi Inoue (2015)** [15]	*	*	*	*	*	*	*	*	**8**
**F Damm (2013)** [6]	*	*	*	*	*	*	*	*	**8**
**F Thol (2013)** [12]	*	*	*	*	*	*	*	*	**8**
**Hideki Makishima (2013)** [5]	*	*	*	*	*	*	*	*	**8**
**Hou (2014)** [10]	*	*	*	*	*	*	*	*	**8**
**M Meggendorfer (2013)** [16]	*	*	*	*	*	*	*	*	**8**
**Michelle A. Elliott (2015)** [7]	*	*			*	*	*	*	**6**
**Mrinal M. Patnaik (2015)** [17]	*	*	*		*	*	*	*	**7**
**RR Laborde (2015)** [11]	*	*	*	*	*	*	*	*	**8**
**Vera Adema (2015)** [18]	*	*	*	*	*	*	*	*	**8**
**Cui (2014)** [19]	*	*	*	*	*	*	*	*	**8**

### Relationship between *SETBP1* mutation and MDS prognosis

**Fig 2:**
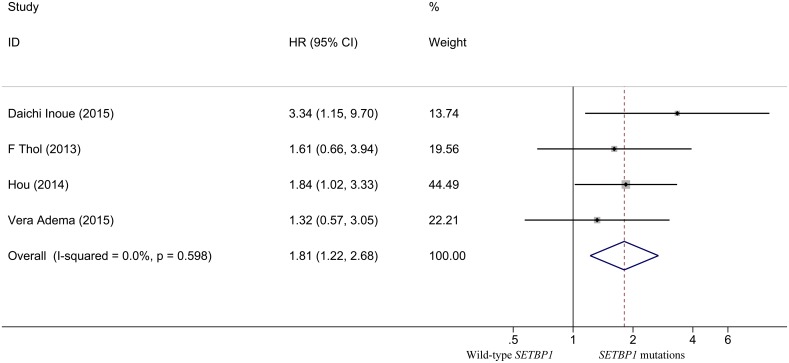
Forest plot of the HR and 95% CI for OS in MDS patients.

**Fig 3:**
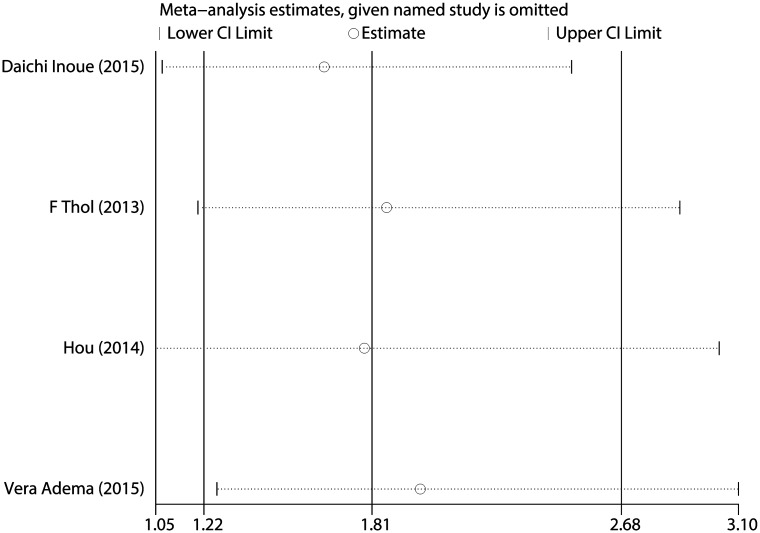
Sensitivity analysis for all included MDS studies.

**Fig 4:**
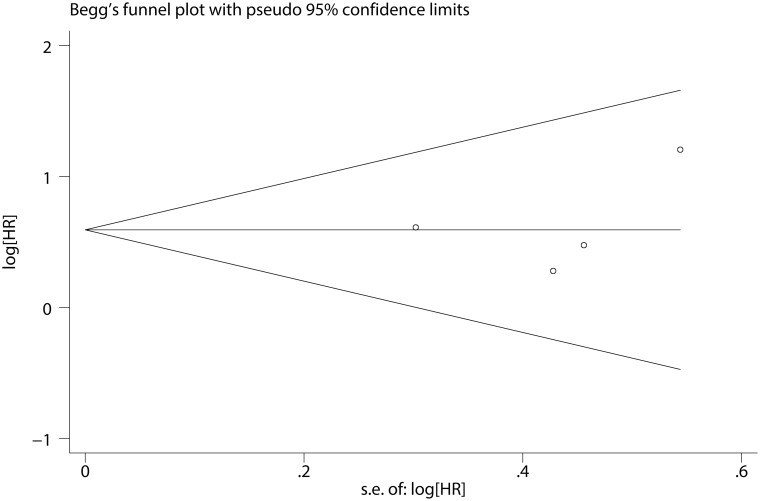
Begg’s funnel plot of the prognostic significance of *SETBP1* mutation in MDS patients.

The quantitative analysis for patients with MDS showed no significant heterogeneity between studies (I^2^ = 0.0%, P = 0.598). Fixed-effects model showed the pooled HR for the OS comparing *SETBP1* mutation versus wild-type *SETBP1* was 1.808 (95% CI: 1.218–2.685, P = 0.001) (Fig 2), suggesting that *SETBP1* mutation may be a factor for the poor prognosis in MDS patients. Sensitivity analysis showed no individual studies significantly change the pooled HR (Fig 3), indicating that the results were reliable and stable. The Begg's (P = 0.734) and Egger's (P = 0.668) tests showed no significant publication bias (Fig 4 and Table 3).

**Table 3:** Meta-analysis of the prognostic effect of SETBP1 mutations compared with wild type *SETBP1* in MDS, CNL and CMML patients.

Study	HR	Lower Limit	Upper Limit	P (HR)	I^2^	P (Heterogeneity)	P (Begg's Test)	P (Egger's test)
CMML	2.223	1.493	3.308	<0.001	30.4%	0.219	0.462	0.568
CNL	1.773	0.877	3.582	0.111	0.0%	0.498	0.296	0.375
MDS	1.808	1.218	2.685	0.001	0.0%	0.598	0.734	0.668

### Relationship between *SETBP1* mutation and CMML prognosis

**Fig 5:**
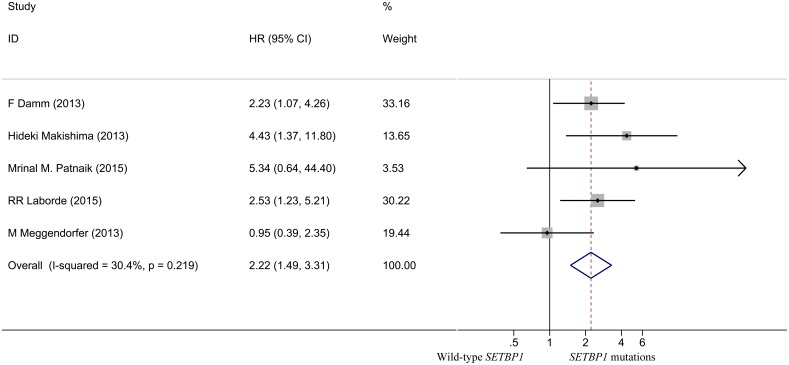
Forest plot of the HR and 95% CI for OS in CMML patients.

**Fig 6:**
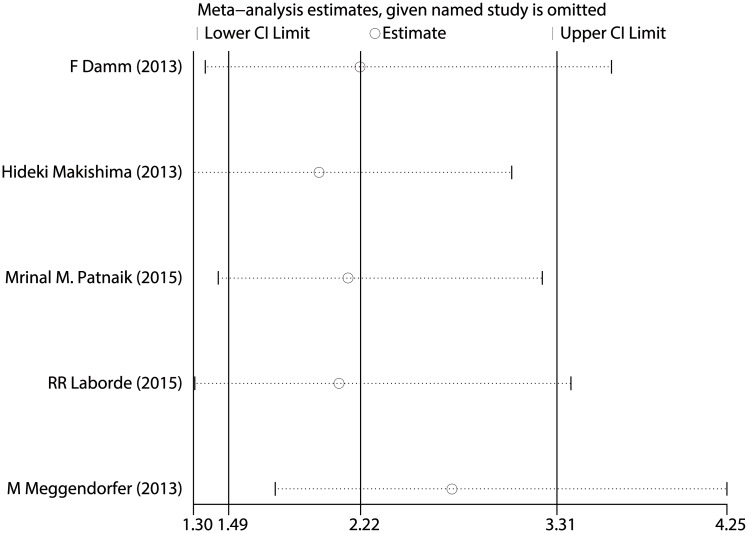
Sensitivity analysis for all included CMML studies.

**Fig 7:**
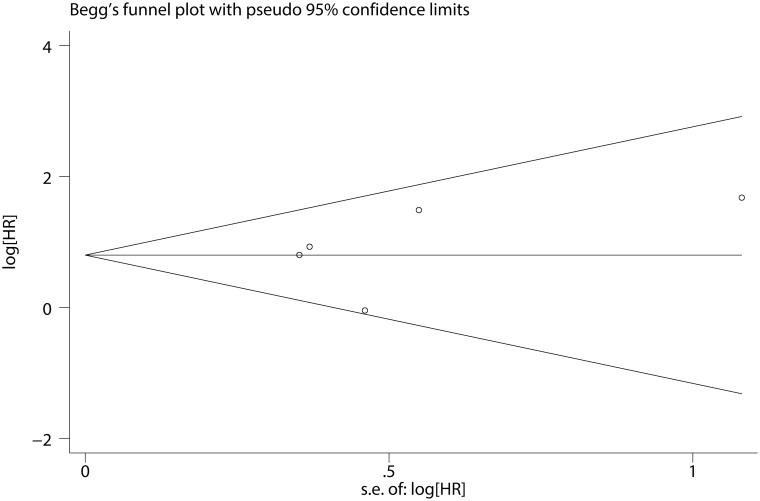
Begg’s funnel plot of the prognostic significance of *SETBP1* mutation in CMML patients.

As shown in Fig 5, the pooled HR for OS in CMML patients was 2.223 (95% CI: 1.493–3.308, P <0.001), suggesting that *SETBP1* mutation may also be a factor of the poor prognosis in CMML patients. No heterogeneity between studies was observed (I^2^ = 30.4%, P = 0.219) and fixed-effects model was applied. Sensitivity analysis showed that the results were consistent (Fig 6). The Begg's (P = 0.462) and Egger's (P = 0.568) tests also revealed no evidence of publication bias (Fig 7 and Table 3).

### Relationship between *SETBP1* mutation and CNL prognosis

**Fig 8:**
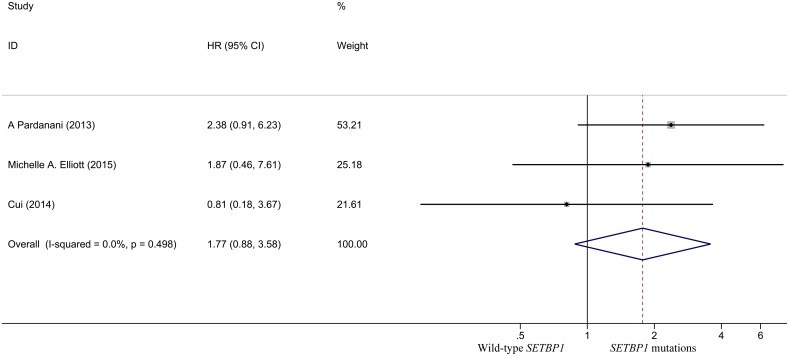
Forest plot of the HR and 95% CI for OS in CNL patients.

**Fig 9:**
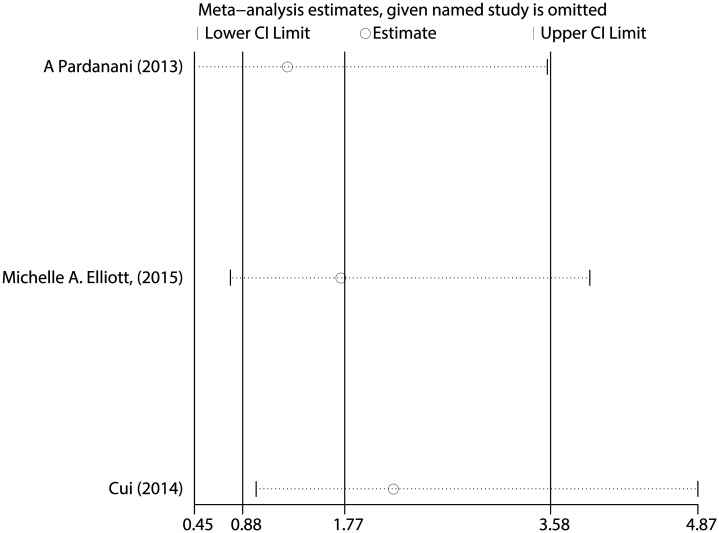
Sensitivity analysis for all included CNL studies.

**Fig 10:**
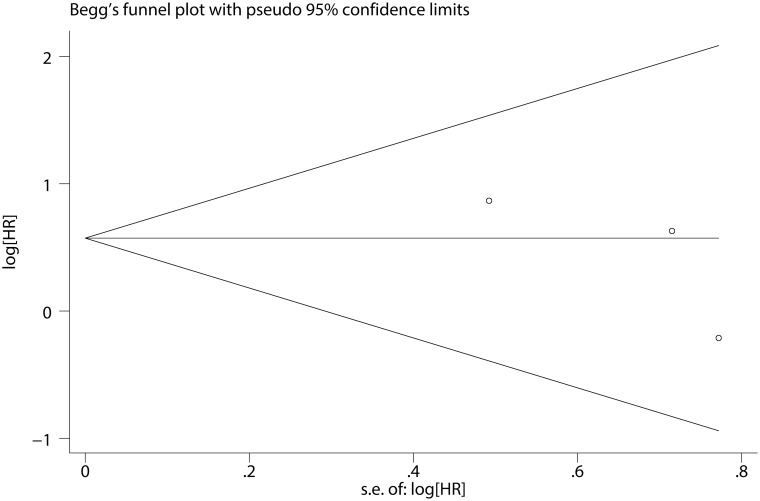
Begg’s funnel plot of the prognostic significance of *SETBP1* mutation in CNL patients.

The pooled HR for the OS in CNL patients was 1.773 (95% CI: 0.877–3.582, P = 0.111) (Fig 8), and showed no statistically significant difference in the prognosis between *SETBP1* mutation and wildtype CNL patients. There was no heterogeneity between studies (I^2^ = 0.0%, P = 0.498) and fixed-effects model was applied. Sensitivity analysis showed that the results were consistent (Fig 9). There was no evidence of publication bias (Begg’s test, P = 0.296; Egger’s test, P = 0.375) (Fig 10 and Table 3).

## Discussion

MDS, CMML, and CNL are chronic myeloid malignancies involved in the hematopoietic system and can be separated into three categories according to the 2008 World Health Organization (WHO) classification: myelodysplastic syndrome (MDS), myeloproliferative neoplasm (CNL), and myelodysplastic/myeloproliferative neoplasms overlap (CMML). These diseases show abnormalities in myeloid progenitor proliferation and differentiation. Genetic mutations are major risk factors for these diseases. Recently, mutations of the *SETBP1* gene have been reported in patients with MDS [10, 12, 15, 18], CMML [5, 6, 11, 16, 17], and CNL [7, 14, 19]. However, the role of *SETBP1* mutations in these diseases remains to be elucidated.

The major aim of this meta-analysis was to investigate the relationship between *SETBP1* mutations and the prognosis in patients with MDS, CMML, or CNL. This meta-analysis suggests that *SETBP1* mutations are strongly associated with a poorer survival in patients with MDS (HR = 1.808, 95% CI (1.218–2.685), P = 0.001) and CMML (HR = 2.223, 95% CI (1.493–3.308), P<0.001), but not in patients with CNL (HR = 1.773, 95% CI (0.877–3.582), P = 0.111). No heterogeneities between studies were observed, indicating that the *SETBP1* mutations are not influenced by variables such as age and sex. Furthermore, Laborde *et al*. has also previously demonstrated that there were no significant correlations between *SETBP1* mutations and age, and gender in CMML patients [11]. Our results show that the prognostic impact of *SETBP1* mutations is similar to that of *ASXL1* mutations, which is one of the strongest independent negative prognostic factor [13, 20] in MDS and CMML patients (HR = 1.45, 95% CI (1.24–1.70)) [21].

Although the role of *SETBP1* remains unclear, Inoue *et al*. recently demonstrated that *SETBP1* mutations augmented leukemic transformation and subsequently adverse survival in MDS patients with *ASXL1* mutations [15]. *SETBP1* mutations targeted the substrate recognition domain of the E3 ubiquitin ligase and inhibited ubiquitin binding [2, 15]. Loss of ubiquitination affected the degradation of SETBP1, leading to a gain-of-function effect. Increased SETBP1 expressions suppressed the PP2A activity, activated the Akt pathway, and enhanced the expression of posterior Hoxa genes in *ASXL1*-mutant cells [15]. This data suggest that *SETBP1* mutations are potential drivers of other mutations that lead to poor prognosis. However, the statistically significant association between *SETBP1* with other mutations is controversial. Damm *et al*. reported that the *SETBP1* mutations observed in about 6% of patients with CMML are likely associated with *ASXL1* mutations [6], while Thol *et al*. demonstrated that no association was found between *ASXL1* and *SETBP1* mutations in his MDS patients [12]. Therefore, this context remains to be determined.

This meta-analysis has a few limitations. First, the relatively small sample sizes of some of the included studies may result in an overestimation of the effect size when compared with larger sample size studies. Second, not all of the HRs with 95% CI values were obtained directly from the included studies. Some studies provided Kaplan-Meier curves rather than the HRs with 95% CI for OS. Therefore, some of the HRs and 95% CI values were obtained from the Kaplan-Meier curves, which might lead to less accurate data. Third, as per discussed, there could be association between *SETBP1* and other mutations. In the current meta-analysis however, we did not demonstrate the association of *SETBP1* mutations with other mutations as there were limited studies available. Moreover, our analyses indicated that were was no heterogeneity between studies and the sensitivity analyses showed the robustness of our results.

## Conclusion

In conclusion, our meta-analysis showed that *SETBP1* mutations have unfavourable prognosis in MDS and CMML patients. These findings may help physicians to evaluate the risk levels and prognosis of the disease for appropriate treatments.

## Supporting information

S1 PRISMA Checklist (DOC)
